# “Misguided Members of the Profession” and the Presidential Addresses Again

**Published:** 1886-11

**Authors:** 


### "MISGUIDED MEMBERS OF THE PROFESSION," AND THE PRESIDENTIAL ADDRESS AGAIN.

### ACTION OF THE EAST LINE (DR. BECTON'S HOME) MEDICAL ASSOCIATION.

GREENVILLE, TEXAS, OCT. 25, 1886.

DR. F. E. DANIEL, Austin, Texas.

*Dear Sir:*—The East Line Medical Association at its last regular meeting held in Greenville, October 5, adopted the enclosed resolutions, and instructed me as Secretary, to furnish them to the *Courier Record of Medicine* and your Texas Journal for publication.

Yours &c.,

J. W. GARNETT, Secretary E. L. M. A.

"WHEREAS, the July number of DANIEL'S MEDICAL JOURNAL contains an article from the editor thereof essaying a criticism upon the several addresses of Drs. Brown and Becton, both having been president of the T. S. M. A., and said article going beyond as we believe, the bounds and spirit of fair and legitimate criticism,

Be it hereby resolved by this Association

*First*, That it is the sense of this Association that the criticism was uncalled for, unfair and undignified, and does not reflect the sentiment of the majority of the physicians of our great State, as claimed—that the language used was opprobrious and personal, and is by us most emphatically condemned.

*Second*, That Drs. Brown and Becton, in the judgment of this body, as presidents severally of T. S. M. A., bore the honors of that position with credit to themselves, and to the approbation of their fellows in medicine.

*Third*, That whereas in the article referred to the editor has assumed to represent the medical fraternity of Texas in matters of theology—we rebuke it as audacious and impertinent.

---

OFFICE DANIEL'S TEXAS MEDICAL JOURNAL,
AUSTIN, TEXAS, October 30, 1886.

DR. J. W. GARNETT, Secretary East Line Medical Association,
Grecnville, Texas.

DEAR SIR: I am in receipt of your letter of October 25, inst., enclosing resolutions passed at a recent meeting of the "East Line Medical Association," censuring me for expressions in the editorial columns of this journal for July.

I regret that you saw proper to withhold them till too late for publication in the October number of the journal, as I should like to have published them simultaneously with the Courier-Record. They shall appear in the next issue of the journal, as requested.

I think your society has taken an extreme view of the matter, and has done me an injustice in construing the article referred to. I allude to the third paragraph of your resolution, which recites that I have "assumed to represent the medical fraternity in matters of theology." I repudiate the charge, and protest, it is not warranted by the facts; that viewed dispassionately, and from an unbiassed standpoint, not a line or sentence in the editorial is legitimately susceptible of any such construction. The Roman tactics of "defending by attacking," which has been attempted by the "East Line Medical Association," is more ingenious than just; for it was *just precisely that assumption,* BY THE PRESIDENTS, (which *you,* and not I, characterize as "audacious and impertinent,")—to which objection was made editorially, as calculated to stir up strife and dissension in the State Association.

Moreover, I have disclaimed (editorially) any intention to reflect personally upon the gentlemen named, and had dismissed the subject, after stating my reason to have been in the interest of harmony in the association; But since the "East Line Medical Association" deline, on behalf of Drs. Becton & Brown, to accept, either my disclaimer or my explanation, but bring up the subject at this late day, and in such shape, I am so clearly put upon the defensive, that I must be excused, if, in my defense, I make use of evidences of approbation, and *endorsement, both of the course and language of the Journal,* quite at pronounced and emphatic as the "censure" of your association. To do this, will, of course, involve a return to the subject in the journal,—something which I had hoped to avoid,—and should the result be,—what *I have sincirely endeavored to prevent,*—feeling and discord among the members of the State Association, I shall hold myself as wholly irresponsible for it.

Very respectfully,

F. E. DANIEL.

(See editorial columns.)

